# Supplementary material for: Mapping a hidden terrane boundary in the mantle lithosphere with lamprophyres
Source: Nat Commun. 2018 Sep 14;9:3770. doi: 10.1038/s41467-018-06253-7 (PMC6138702; doi:10.1038/s41467-018-06253-7)
Supplement: Supplementary file 1 — Supplementary Information [file 41467_2018_6253_MOESM1_ESM.pdf]

Supplementary Information

Mapping a hidden terrane boundary in the mantle lithosphere with lamprophyres

Arjan H. Dijkstra & Callum Hatch

Contains:

Supplementary Figures 1-4

Supplementary Table 1

Supplementary References

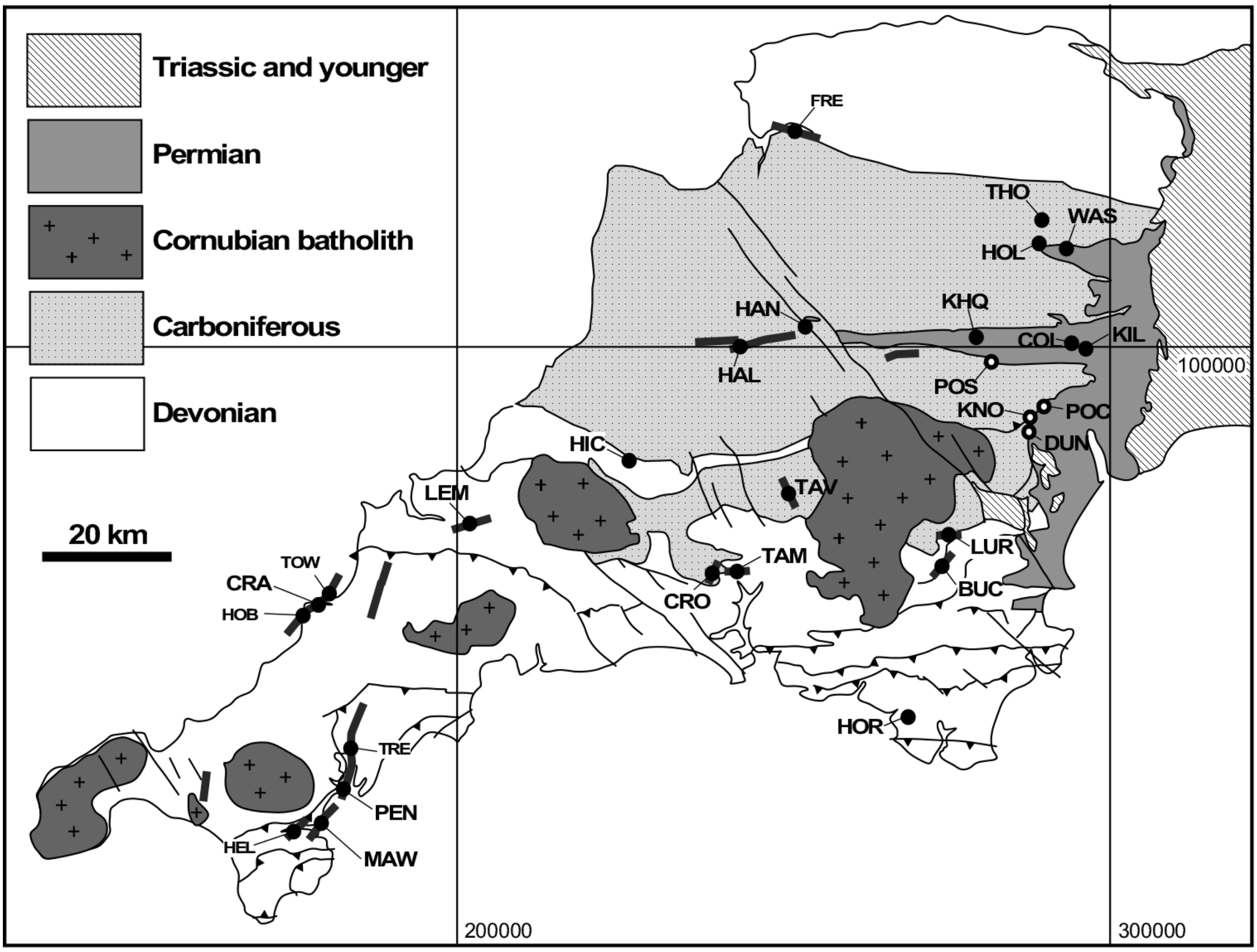

Supplementary Figure 1. Distribution of lamprophyres and locations discussed in the text and figures. Lamprophyres shown as filled circles; high-K lavas shown as open circles. In addition to data from the present study, published data[1] from lamprophyre localities HEL (Helford), Trelissick (TRE), HOB (Holywell Beach), TOW (Towan Head) and FRE (Fremington Quay) is also used in the analysis. Ordnance Survey British Grid Coordinates shown. Map adapted from regional view geological map from British Geological Survey[2]. © Crown Copyright and Database Right 2018. Ordnance Survey (Digimap Licence).

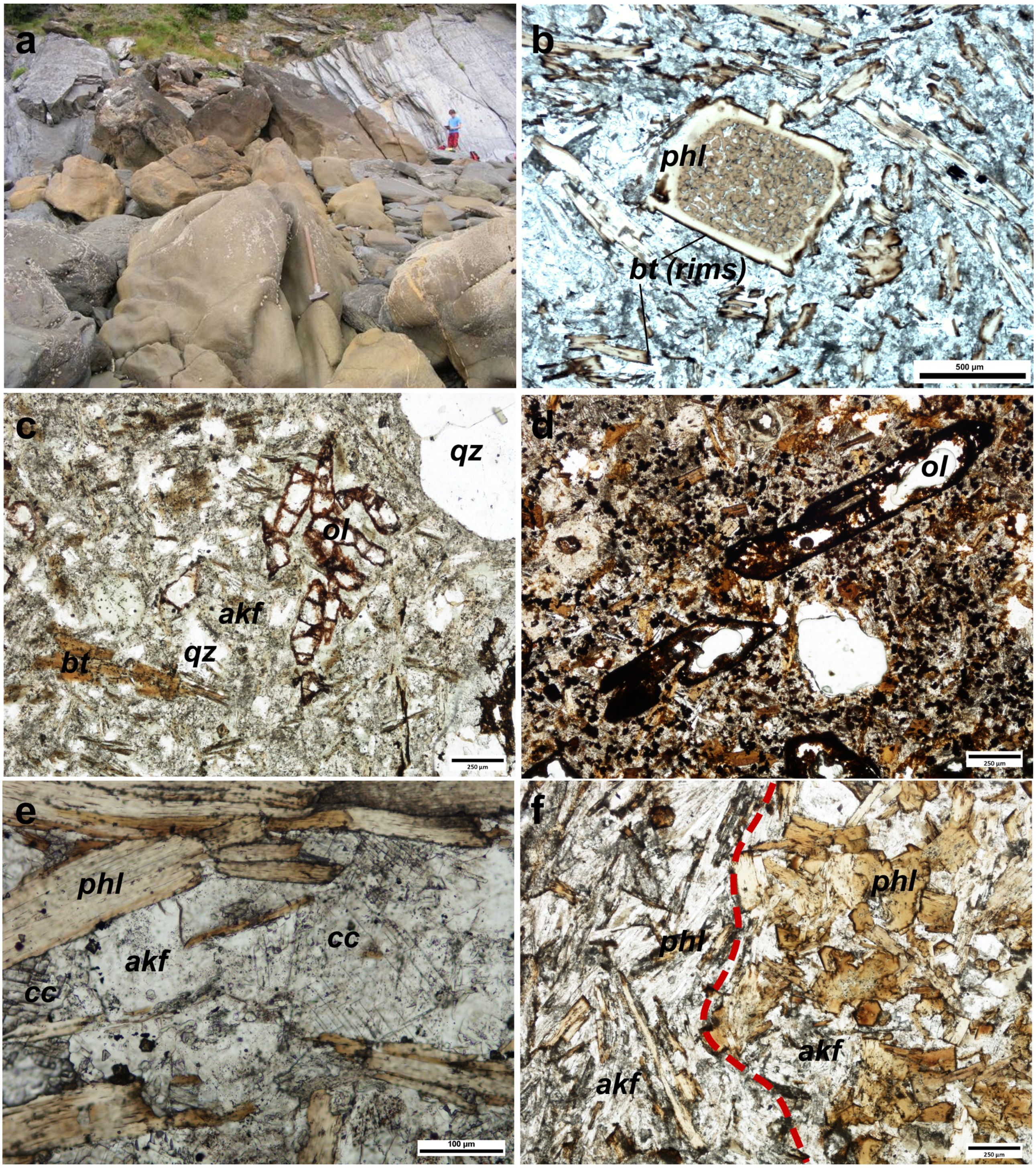

Supplementary Figure 2. a) Orange-brown lamprophyre exposure within light grey Devonian slates at Mawnan (MAW). Hammer shaft (c. 70 cm) for scale. b) Zones dark mica phenocrysts with pale phlogopite (phl) cores and dark biotite (bt) rims. Large phenocryst in centre has a sieve-like texture, sample MAW; c) Minette consisting of phlogopite (phl), altered alkali-feldspar (akf), and quartz (qz), with serpentinized olivine (ol) phenocrysts and a rounded quartz xenocryst in top right corner, sample BUC; d) skeletal embayed olivine phenocrysts in minette from WAS; e) Close-up of minette containing dolomitic calcite (cc, with rhombohedral cleavage) in addition to phlogopite and alkali-feldspar, sample PEN; f) Phlogopite-rich cumulate inclusion in minette from sample MAW.

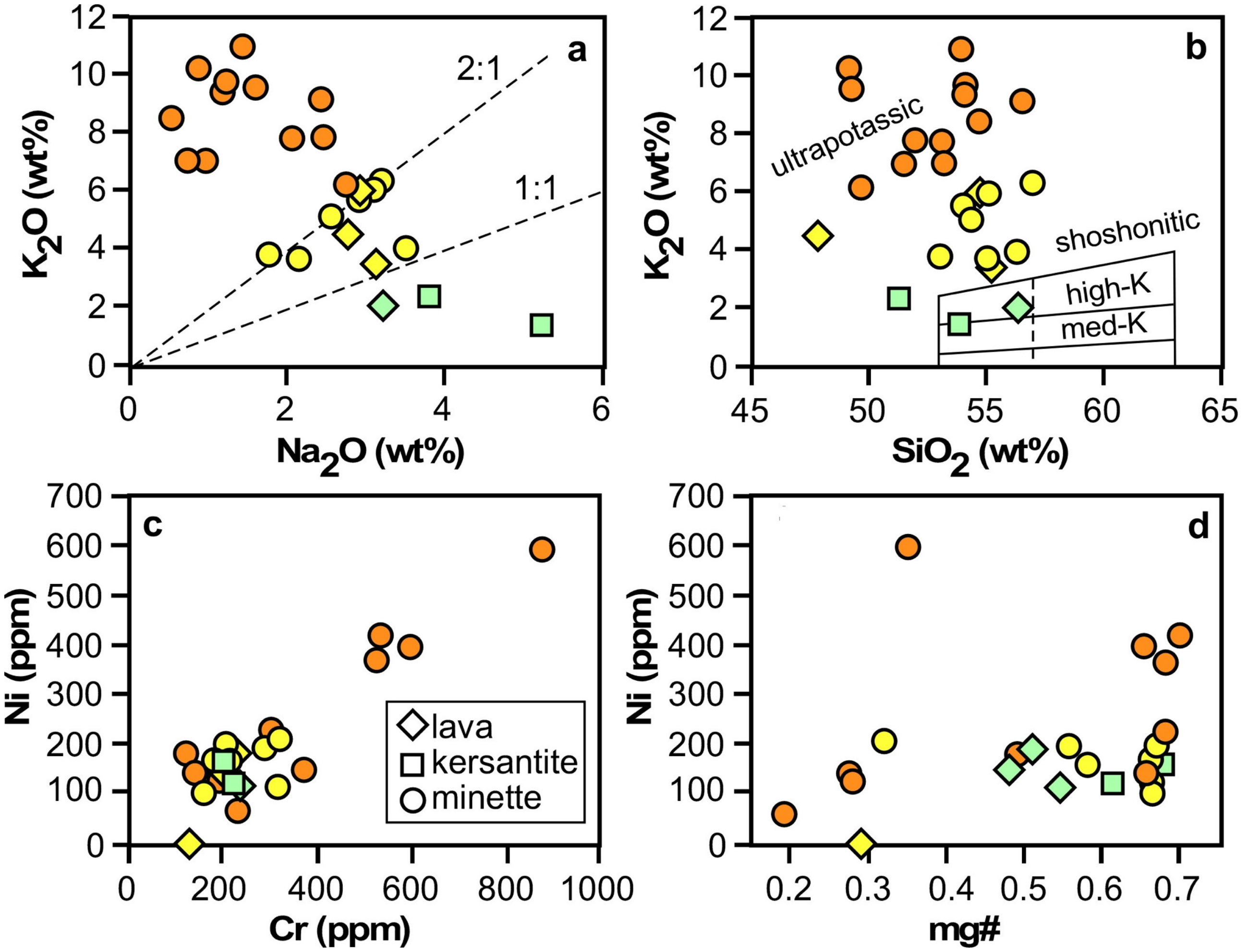

Supplementary Figure 3. Diagrams showing key geochemical indicators for lamprophyres (minettes as circles, and kersantites as squares) and high-K lavas (diamonds) from southwest Britain. Concentrations of major element oxides recalculated on an anhydrous basis and with all iron reported as FeO. a) Na_2O-K_2O diagram (wt%). For internal consistency of groupings in diagrams in panels a and b, $K_2O/Na_2O>2.2$ is chosen as the lower boundary of the ultrapotassic group. Ultrapotassic rocks in orange, potassic rocks in yellow and high-K rocks with $K_2O/Na_2O<1$ in green. b) SiO_2-K_2O (wt%) classification diagram for potassic magmas. c) Concentrations of compatible elements Cr and Ni (ppm) showing the generally primitive nature of the parental magmas. d) Mg-number (mg#) versus Ni (ppm), where mg# is the atomic ratio Mg/(Mg+Fe). Rocks with mg# approaching 0.70-0.74 are deemed to have formed from near-primary mantle melts.

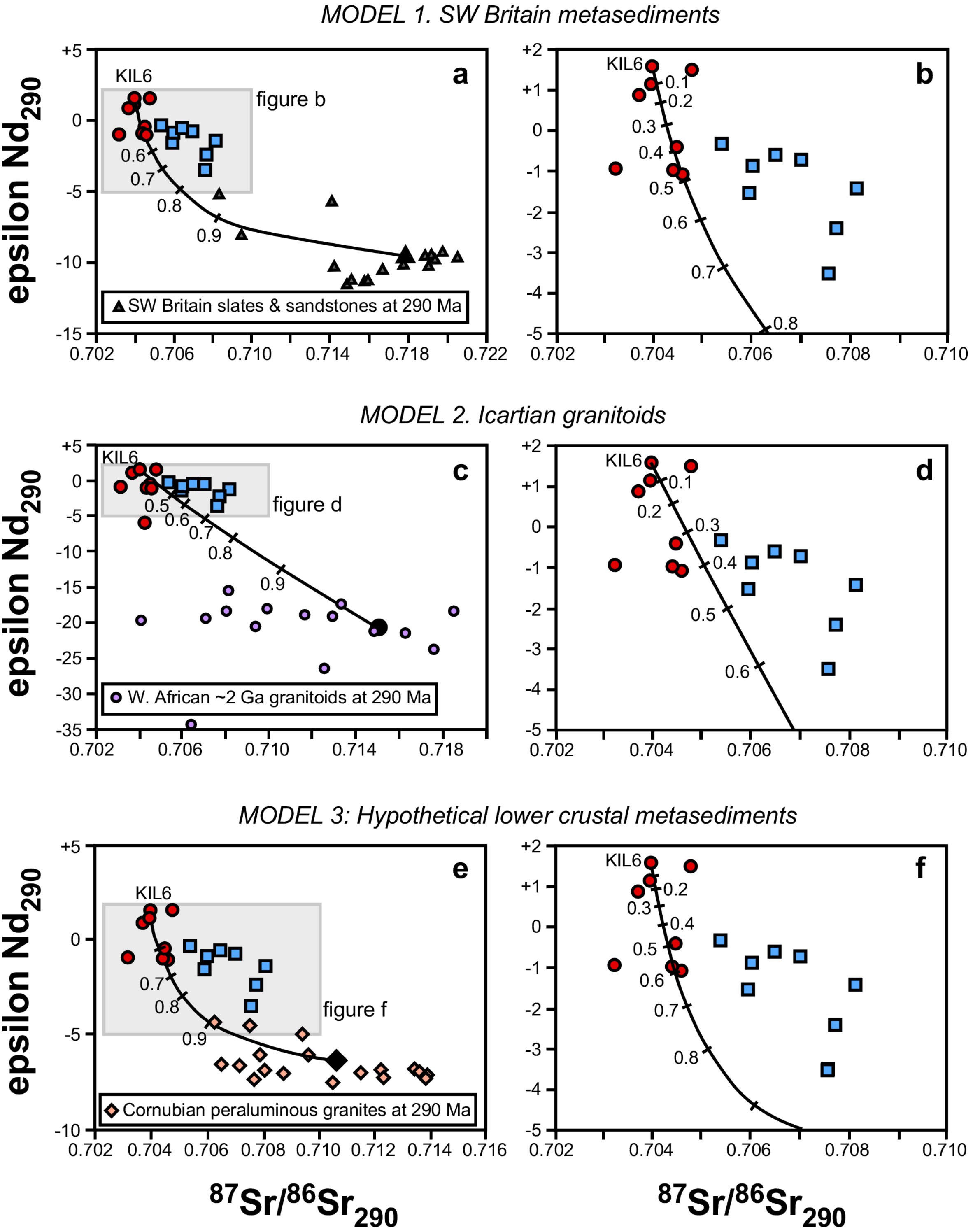

Supplementary Figure 4. Mixing models to assess effect of contamination of lamprophyre magmas with crustal material during emplacement. Group 1 rocks shown as red circles, group 2 rocks shown as blue squares. Values near ticks represent fractions (0<X<1) of contaminant in the mixture (e.g., X=0.5 is a mixture of 50% KIL6 magma with 50% contaminant). Model 1 in panels a-b uses an average metasediment from southwest Britain[3] as the assimilated crustal contaminant. Model 2 in panels c-d uses a typical 2 Ga Icartian granitoid such as found on the Channel Islands[4] and in West Africa[5,6] as a model for typical Armorican meta-igneous basement components in the lower crust of southwest Britain as potential contaminants. Model 3 in panels e-f uses a hypothetical lower crustal metasediment, with an isotopic composition corresponding to that of a typical peraluminous (probably sediment-derived) Permian granite from the Cornubian Batholith at 290 Ma[3] and Rb/Sr and Sm/Nd ratios similar to those of typical metasediments as in model 1.

Supplementary Table 1. Sample localities and accession numbers for samples deposited at Plymouth Museum

		Easting (m)	Northing (m)	*Museum Accession no.*
lamprophyres				
**THO**	Thorne Farm (loose sample from end of driveway)	289684	119024	*PLYMG.2018.79.1*
**HOL**	Holmead Farm (loose sample from hedgerow)	289266	115739	*PLYMG.2018.79.2*
**WAS**	Washfield (loose sample from back-filled quarry)	293138	115426	*PLYMG.2018.79.3*
**KHQ**	Knowle Hill Quarry (in-situ, collected during excavation)	278998	102218	*PLYMG.2018.79.4*
**HAL**	Hallwill Junction (loose sample from old quarry)	242704	100044	*PLYMG.2018.79.5*
**KIL**	Killerton House (loose sample from old quarry)	297500	100483	*PLYMG.2018.79.6*
**COL1**	Columbjohn Wood (loose sample from old quarry)	295964	99896	*PLYMG.2018.79.7*
**COL3**	Columbjohn Wood (loose sample from old quarry)	295964	99896	*PLYMG.2018.79.8*
**HIC**	Hick's Mill (PU research collection)	225988	82946	*PLYMG.2018.79.9*
**TAV**	Peter Tavy (in-situ, W bank of river Tavy)	250790	77520	*PLYMG.2018.79.10*
**LEM**	Lemail (loose sample from railway cut)	202231	73103	*PLYMG.2018.79.11*
**LUR**	Lurgecombe (loose sample from back-filled quarry)	275573	71362	*PLYMG.2018.79.12*
**BUC**	Buckfastleigh (in-situ sample from new roadcut)	273596	66566	*PLYMG.2018.79.13*
**TAM**	Tamar (loose sample from bank of river)	242350	65952	*PLYMG.2018.79.14*
**CRO**	Crocadon Wood (loose sample from logging road cut)	239372	65550	*PLYMG.2018.79.15*
**CRA**	Crantock, Newquay (little beach, in-situ)	179308	61060	*PLYMG.2018.79.16*
**HOR**	Horswell House (old quarry, loose sample)	268761	42141	*PLYMG.2018.79.17*
**PEN**	Pendennis Point, Falmouth (in-situ sample foreshore)	182755	31525	*PLYMG.2018.79.18*
**MAW**	Mawnan (foreshore, loose sample western dyke)	178860	27172	*PLYMG.2018.79.19*
**MAW-CB2**	Mawnan (foreshore, loose sample eastern dyke)	179135	27218	*PLYMG.2018.79.20*
K-rich lavas				
**POS**	Posburry Clumb (in-situ sample from old quarry)	281433	97846	*PLYMG.2018.79.21*
**POC**	Pocombe, Exeter (in-situ sample from roadcut)	289885	91247	*PLYMG.2018.79.22*
**KNO**	Knowle (loose sample from old quarry)	287426	89547	*PLYMG.2018.79.23*
**DUN**	Dunchideock (in-situ sample from old quarry)	287588	87211	*PLYMG.2018.79.24*
Basaltic dyke from Lizard Ophiolite (c. 380 Ma) for comparison in figure 3				
**COV**	Coverack beach	178362	18709	

Coordinates: Ordnance Survey British Grid

Supplementary References

1. Dupuis, N.E., Murphy, J.B., Braid, J.A., Shail, R.K., & Nance, R.D. Mantle evolution in the Variscides of SW England: Geochemical and isotopic constraints from mafic rocks. *Tectonophysics* **681**, 353-363 (2016).
2. British Geological Survey. Geological Map Data BGS (2010)
3. Darbyshire, D.P.F. & Shepherd, T.J. Nd and Sr isotope constraints on the origin of the Cornubian batholith, SW England. *Journal of the Geological Society* **151**, 795-802 (1994).
4. Samson, S.D. & RS D'Lemos, R.S. U-Pb geochronology and Sm-Nd isotopic composition of Proterozoic gneisses, Channel Islands, UK. *Journal of the Geological Society London* **155**, 609-618 (1998).
5. Boher, M., Abouchami, W., Michard, A., Albarede, F. & Arndt, N.T. Crustal growth in West Africa at 2.1 Ga. *Journal of Geophysical Research* **97**, 345–369 (1992).
6. Gasquet, D., Barbey, P., Adou, M. & Paquette, J.L. Structure, Sr-Nd isotope geochemistry and zircon U-Pb geochronology of the granitoids of the Dabakala area (Côte d'Ivoire): evidence for a 2.3 Ga crustal growth event in the Palaeoproterozoic of West Africa? *Precambrian Research* **127**, 329-354 (2003).
