# Supplementary material for: Mapping a hidden terrane boundary in the mantle lithosphere with lamprophyres
Source: Nat Commun. 2018 Sep 14;9:3770. doi: 10.1038/s41467-018-06253-7 (PMC6138702; doi:10.1038/s41467-018-06253-7)
Supplement: Supplementary file 3 — Description of Additional Supplementary Files [file 41467_2018_6253_MOESM3_ESM.pdf]

Description of additional supplementary files

File name: Supplementary Data 1

Description: Excel file containing major element data (XRF), trace and minor element data (ICP-MS) and Sr and Nd isotope data (TIMS & MC-ICP-MS) of bulk rock samples discussed in the paper. Second worksheet contains major element data for Certified Reference Materials determined by XRF.
